# Video head impulse test for the assessment of vestibular function in patients with idiopathic sudden sensorineural hearing loss without vertigo

**DOI:** 10.1017/S0022215123000245

**Published:** 2023-12

**Authors:** N Battat, O J Ungar, O Handzel, R Abu Eta, Y Oron

**Affiliations:** 1 Department of Otolaryngology – Head and Neck Surgery, Samson Assuta Ashdod University Hospital, Ben Gurion University Faculty of Health Sciences, Ashdod, Israel; 2 Department of Otolaryngology Head and Neck Surgery and Maxillofacial Surgery, Tel Aviv Sourasky Medical Center, Tel Aviv, Israel; 3 Faculty of Medicine, Tel Aviv University, Tel Aviv, Israel

**Keywords:** Sensorineural hearing loss, vertigo, dizziness, head impulse test

## Abstract

**Objective:**

Idiopathic sudden sensorineural hearing loss may be accompanied by dizziness without true vertigo. This study used the video head impulse test to evaluate vestibular function in idiopathic sudden sensorineural hearing loss patients who described experiencing dizziness and not true vertigo.

**Methods:**

A prospective study was conducted of 30 consecutive patients diagnosed with idiopathic sudden sensorineural hearing loss with dizziness without true vertigo. A comparison of the video head impulse test results of the patients who complained of dizziness (symptomatic group) with a group of patients with idiopathic sudden sensorineural hearing loss and no dizziness (asymptomatic) was performed.

**Results:**

Nine patients (30 per cent) were symptomatic. Two of those patients had abnormal video head impulse test findings. Seven patients in the asymptomatic group (7 out of 21, 33 per cent) presented with abnormal video head impulse test results. No significant difference in vestibular function between the two groups was detected by the video head impulse test.

**Conclusion:**

The site of insult in patients with idiopathic sudden sensorineural hearing loss without true vertigo is usually limited to the cochlea or the cochlear nerve.

## Introduction

Idiopathic sudden sensorineural hearing loss (SNHL) is defined as a hearing loss of more than 30 dB in at least three contiguous audiometric frequencies and occurring over a 72-hour period.^1^ The overall annual incidence of idiopathic sudden SNHL is approximately 25 per 100 000 individuals, and it has been found to increase with age.^2^ Several aetiologies of idiopathic sudden SNHL, such as viral infection, cochlear membrane breaks and vascular insult, have been suggested, but the exact cause and mechanism are still unknown.^3^

Vertigo is a symptom of vestibular dysfunction, described as a sensation of motion, most commonly rotational.^4^ Vestibular involvement in idiopathic sudden SNHL was first reported in 1949, and its incidence was estimated as being between 30 and 40 per cent.^5–7^ A significant percentage of patients diagnosed with idiopathic sudden SNHL complain of mild-to-moderate instability,^8^ but no evidence of true vertigo is detected by the physical examination for vestibular function in most cases. Although vestibular dysfunction in idiopathic sudden SNHL is hypothesised to be an extension of the condition from the cochlea to the vestibular organs given their anatomical proximity, the relationship and pathogenesis of cochlear dysfunction and vestibular involvement in this setting remain controversial.^9^

In addition to that, it is common to see patients with idiopathic sudden SNHL who describe non-vertiginous dizziness; that is, a sensation of disturbed or impaired spatial orientation without a false or distorted sense of motion.^10^ The vestibular function in cases of idiopathic sudden SNHL with dizziness but without true vertigo has not been specifically investigated.

The video head impulse test, which evaluates the gain of the vestibulo-ocular reflex of each semicircular canal, is easy to perform, safe and comfortable for the patient. The data published thus far have highlighted the superiority of the video head impulse test in routine vestibular assessment and in the identification of vestibular weakness through accurate measurement of reduced vestibulo-ocular reflex gain.^11,12^

The present study aimed to evaluate the vestibular function in idiopathic sudden SNHL patients who do not report true vertigo, using the video head impulse test.

## Materials and methods

### Patients

We conducted a prospective study between January 2020 and August 2021 in which all patients older than 18 years of age who were admitted to the Department of Otolaryngology – Head and Neck Surgery at a tertiary medical centre with the diagnosis of idiopathic sudden SNHL underwent a vestibular evaluation by means of the video head impulse test at the time of admission.

All of the enrolled patients were diagnosed as having idiopathic sudden SNHL, which was defined as a hearing loss of more than 30 dB over three contiguous frequencies and occurring within 72 hours.^8^ We excluded patients who described a sensation of motion – that is, true vertigo – but included patients who described non-vertiginous dizziness.^10^ Other exclusion criteria were: vestibular dysfunction due to other identifiable causes, patients who were unable to undergo video head impulse testing because of neck pain or reduced neck range of motion, patients with previous known chronic vestibular dysfunction, patients treated with medications that might affect their vestibular function, and pregnant women.

The evaluations included general and audiovestibular medical history-taking, physical examination, pure tone audiometry (Interacoustics AC40 clinical audiometer) and the video head impulse test (Interacoustics Eclipse system), which were conducted prior to initiation of treatment. All results were recorded and the data were completely anonymised before the analyses. All the participants had given their written informed consent. The study was approved by the institutional review board (protocol number: 633-19).

### Video head impulse test recording procedure

The video head impulse test was performed by a single physician who was not blinded to the participants’ symptoms. A high-speed video camera with an embedded accelerometer and a gyrometer in the tightly fitting video goggles recorded the eye movements (right eye) with respect to the head movement. The visual target at the eye level was located on the wall at a distance of 1 m. Calibration (eye position) was carried out by asking the patients to look through the goggles at laser dots projected on the wall. The dots were presented arbitrarily, and the patients were instructed to follow them. The patients were instructed to maintain their gaze on the visual target, to keep their neck muscles relaxed and to keep their eyes wide open with minimal blinking. The examiner stood behind the patients and rotated their head in three planes, corresponding with the three semicircular canals on both sides, using fast unpredictable rotational impulses of the head (100–300 degrees per second) with a low amplitude (less than 20 degrees).^13^ At least 14 impulses were given, and the responses were averaged in order to obtain the mean vestibulo-ocular reflex gain.

### Video head impulse test outcome parameters

The parameters of interest were the vestibulo-ocular reflex gains for the three planes on each side after head movement and the presence of corrective saccades. Any asymmetry of vestibulo-ocular reflex gain between the ears, an abnormally high or low gain, and the presence of corrective saccades were considered abnormal results.

## Results

The 30 patients who comprised the study group included 15 males and 15 females, with a mean age of 56 years (range, 24–86 years). Twelve patients (40 per cent) were healthy adults with no relevant medical history, and two patients reported a single event of benign positional paroxysmal vertigo that had occurred years before the idiopathic sudden SNHL. The average time from onset of hearing symptoms to treatment was 6.4 days (range, 1–21 days). The clinical characteristics of these 30 patients are presented in Table 1.

**Table 1.** Patients’ clinical characteristics

Characteristic	Patients (*n* (%))
Co-morbidities	
– Essential hypertension	12 (40)
– Diabetes mellitus type 2	3 (10)
– Hypothyroidism	2 (6.7)
– Crohn's disease	1 (3.3)
– None	12 (40)
Hearing loss severity in 3 contiguous frequencies	
– Mild (25–40 dB)	2 (6.7)
– Moderate (40–55 dB)	12 (40)
– Moderate–severe (55–70 dB)	11 (36.7)
– Severe (>70 dB)	5 (16.7)
Associated symptoms*	
– Dizziness	9 (30)
– Tinnitus	27 (90)
– Ear fullness	28 (93.3)

* Some patients had more than one symptom

Twenty-one of the 30 patients (70 per cent) did not describe any vestibular symptoms (asymptomatic group), while 9 of the patients (30 per cent) described dizziness (but not vertigo) at some point in proximity to the detection of the hearing loss (symptomatic group). Six of these nine patients were among those who had reported dizziness at the onset of hearing impairment. They all reported spontaneous resolution of the symptoms within 1 hour. One of those patients complained of a sensation of chronic instability throughout the past two years and experienced dizziness during the examination. Two other patients reported sensations of intermittent dizziness that lasted for a few minutes and that recurred once or twice a day beginning shortly before the onset of the hearing impairment. All of those six patients had unremarkable physical examination findings, with no signs of vestibular or neurological dysfunction. Nine other patients (30 per cent) had abnormal findings on the video head impulse test, of whom six were asymptomatic and three were symptomatic.

The patterns of video head impulse test abnormalities were as follows. Three patients had overt or covert saccades, two of whom were asymptomatic and the third complained of transient dizziness that had resolved before the video head impulse test was conducted. Nine patients (23 per cent) had abnormal vestibulo-ocular reflex gain (two from the symptomatic group and seven from the asymptomatic group). Two of those nine patients had asymmetric low gain (below 0.6) in a single canal, and one patient had low gain in all six canals. The latter patient did not have vestibular symptoms or corrective saccades. Three patients had an abnormal and asymmetric high gain (above 1.5) in one canal only, and one patient had an abnormally high gain of 2.7 in two posterior canals.

The abnormal video head impulse test results were similar in both the symptomatic group and the asymptomatic group. Two of the nine patients in the symptomatic group (22 per cent) had an abnormal vestibulo-ocular reflex gain in one plane, one with high gain (1.7) and the other with both low gain (0.37) and corrective saccades; neither patient had vestibular symptoms at the time of the video head impulse test. Seven of the 21 patients in the non-symptomatic group (33 per cent) had abnormal video head impulse test findings: 2 patients (9.5 per cent) had corrective saccades and 5 (24 per cent) had an abnormal vestibulo-ocular reflex gain (Table 2). A chi-square test of independence, performed to examine the relation between the presence of symptoms and the video head impulse test results, revealed no significant difference in the rate of abnormal video head impulse test results between the two groups (χ^2^ = 0.37; *p* > 0.05).

**Table 2.** Summary of vestibular symptoms and findings on VHIT

Variable	Asymptomatic patients*	Symptomatic patients^†^	All patients^‡^
Corrective saccades	2	1	3
Asymmetric high gain	2	1	3
Asymmetric low gain	1	1	2
Symmetric low gain	1	0	1
Symmetric high gain	1	0	1
Total VHIT findings	7	3	10

Data represent numbers of patients. *n = 21; ^†^*n* = 9; ^‡^*n* = 30. VHIT = video head impulse test

## Discussion

This is the first study to assess the rate of vestibular dysfunction specifically among patients with idiopathic sudden SNHL without vertigo, using the video head impulse test.

Several earlier studies sought to detect the site of vestibular insult in idiopathic sudden SNHL patients by means of vestibular function tests, including videonystagmography or electronystagmography, and cervical or ocular vestibular-evoked myogenic potentials, and found that the posterior semicircular canal was the most frequently affected vestibular end-organ in idiopathic sudden SNHL.^14–17^ More recent reports demonstrated that the involvement of the posterior semicircular canal was associated with poor prognosis of hearing recovery.^15,18^ Those studies, however, did not differentiate between patients with and without true vertigo, and some did not use the video head impulse test as a measure of vestibular function.

A retrospective study by Seo *et al*. investigated the patterns of semicircular canal and otolith organ dysfunction in idiopathic sudden SNHL by analysing cervical or ocular vestibular-evoked myogenic potential results and video head impulse test results.^18^ In that study, the vestibular end-organ showed various patterns of dysfunction in patients with idiopathic sudden SNHL. Those authors reported a rate of 22.2 per cent for posterior semicircular canal impairment and a rate of 7.4 per cent each for the horizontal and the anterior canals.^18^ These rates of impairment are higher than the total rate of 33 per cent found in the current study. Their study also included patients with and without vertigo; therefore, their results represent a different type of patient population among whom the semicircular canals are affected, given that it is reflected in the patients’ vertigo symptoms.

In a study published in 2022,^19^ the vestibular function of patients with idiopathic sudden SNHL was studied using videonystagmography, caloric study and vestibular-evoked myogenic potential. Abnormal caloric response was found to be higher in patients with profound hearing loss. However, in that study, there was no discrimination between patients with and without vertigo regarding the abnormal caloric response outcome, and canal function was not measured using the video head impulse test.

In contrast to previous studies, our study aimed to assess the vestibular end-organ function in patients with idiopathic sudden SNHL who complained of dizziness but not true vertigo. One of the key components in vestibular assessment is the measurement of the vestibulo-ocular reflex gain. The video head impulse test measures the vestibulo-ocular reflex by measuring the function of the semicircular canal. We compared the video head impulse test results of patients with dizziness (symptomatic group) with those of patients with idiopathic sudden SNHL and neither dizziness nor vertigo (asymptomatic group), to detect subtle vestibular dysfunction patterns that might explain patients’ complaints.

Nine of our study patients had abnormal video head impulse test results, and six others were free of vestibular symptoms. This may be the result of a subclinical insult to the vestibular organ or an old vestibular insult that was completely compensated for by the time the video head impulse test was conducted, and therefore did not cause any vestibular sensations. Only one patient in our study who complained of subjective transient dizziness had an abnormal gain in the video head impulse test result, specifically an asymmetric low gain (of 0.37) in the right-lateral semicircular canal. The rate of abnormal video head impulse test results was not statistically different between the symptomatic and asymptomatic patient groups. We therefore conclude that vestibular dysfunction was not detected by the video head impulse test in most patients with idiopathic sudden SNHL with or without vestibular symptoms. These results imply that despite subjective dizziness symptoms among patients with idiopathic sudden SNHL, in most cases no objective insult can be detected by the video head impulse test. However, we did find that asymptomatic patients (without vertigo or dizziness) may display abnormal video head impulse test results.

- A significant percentage of patients diagnosed with idiopathic sudden sensorineural hearing loss (SNHL) complain of mild-to-moderate instability
- The vestibular function in cases of idiopathic sudden SNHL with dizziness but without true vertigo has not been specifically investigated
- The video head impulse test evaluates the vestibulo-ocular reflex gain of each semicircular canal
- Vestibular dysfunction was not detected by the video head impulse test in most patients with idiopathic sudden SNHL with or without vestibular symptoms
- In idiopathic sudden SNHL, the insult site is usually isolated to the cochlea or cochlear nerve, without vestibular involvement

This study has some limitations. It includes a small sample size of 30 patients, and we did not perform other vestibular tests, such as cervical or ocular vestibular-evoked myogenic potentials, which might have assisted in correlating the patients’ symptoms with vestibular dysfunction. In addition, the physician who performed the video head impulse test was not blinded to the participants’ symptoms, a fact that might have affected the test results. Further studies may correlate these test findings with the patients’ hearing prognosis and a control group of patients with vertigo.

## Conclusion

We applied the video head impulse test to examine vestibular manifestations in idiopathic sudden SNHL patients with symptoms of subjective dizziness, without true vertigo. Our findings suggest that the site of insult is usually isolated to the cochlea or the cochlear nerve in such cases, without vestibular involvement.
